# Targeted therapy of cancer stem cells: inhibition of mTOR in pre-clinical and clinical research

**DOI:** 10.1038/s41419-024-07077-8

**Published:** 2024-09-30

**Authors:** Boram Son, Wonhwa Lee, Hyeonjeong Kim, Heungsoo Shin, Hee Ho Park

**Affiliations:** 1 Department of Bioengineering, Hanyang University, Seoul, 04763 Republic of Korea; 2 Department of Bio and Fermentation Convergence Technology, Kookmin University, Seoul, 02707, Republic of Korea; 3 Department of Chemistry, Sungkyunkwan University, Suwon, 16419 Republic of Korea; 4 Research Institute for Convergence of Basic Science, Hanyang University, Seoul, 04763 Republic of Korea

**Keywords:** Cancer stem cells, Targeted therapies

## Abstract

Cancer stem cells (CSCs) are a type of stem cell that possesses not only the intrinsic abilities of stem cells but also the properties of cancer cells. Therefore, CSCs are known to have self-renewal and outstanding proliferation capacity, along with the potential to differentiate into specific types of tumor cells. Cancers typically originate from CSCs, making them a significant target for tumor treatment. Among the related cascades of the CSCs, mammalian target of rapamycin (mTOR) pathway is regarded as one of the most important signaling pathways because of its association with significant upstream signaling: phosphatidylinositol 3‑kinase/protein kinase B (PI3K/AKT) pathway and mitogen‑activated protein kinase (MAPK) cascade, which influence various activities of stem cells, including CSCs. Recent studies have shown that the mTOR pathway not only affects generation of CSCs but also the maintenance of their pluripotency. Furthermore, the maintenance of pluripotency or differentiation into specific types of cancer cells depends on the regulation of the mTOR signal in CSCs. Consequently, the clinical potential and importance of mTOR in effective cancer therapy are increasing. In this review, we demonstrate the association between the mTOR pathway and cancer, including CSCs. Additionally, we discuss a new concept for anti-cancer drug development aimed at overcoming existing drawbacks, such as drug resistance, by targeting CSCs through mTOR inhibition.

## Facts

- Cancer treatment faces challenges due to its complexity, with chemotherapy limited by drug resistance, prompting the exploration of advanced therapeutic approaches.
- Cancer Stem Cells (CSCs) are a key focus, sharing characteristics with normal stem cells and being targeted to prevent differentiation into tumor cells and promote self-renewal.
- The mTOR pathway plays a pivotal role in activating stem and immune cells within cancer tissues, leading to the development and utilization of mTOR inhibitors in FDA-approved cancer treatments.
- Clinical trials categorize mTOR inhibitors for various cancer types, demonstrating their efficacy in suppressing CSC differentiation, intrinsic stem cell properties, and reducing metastasis.
- Despite promising results, caution is warranted in mTOR inhibition due to its involvement in normal cellular activities, necessitating careful strategies to avoid potential side effects and the development of advanced, targeted tumor therapies.

## Open questions

- How can CSC-targeting therapy effectively prevent tumor recurrence, considering the pivotal role of CSCs in tumor relapse, metastasis, and resistance to radiotherapy and chemotherapy?
- What intrinsic factors contribute to the development of resistance in CSCs, including their longer cell cycle, DNA repair system, and involvement of oxidative modulators and metabolic plasticity regulators?
- In what ways does the PI3K/AKT/mTOR axis contribute to CSC resistance, and what are the outcomes and implications of clinical trials involving mTOR pathway inhibitors for various cancers?
- What challenges are associated with delivering anti-cancer drugs to CSCs, and how effective are viral delivery systems like adenovirus and nanoparticle therapeutics in reaching and targeting CSCs, considering existing challenges in these delivery methods?

## Introduction

Stem cells are known for their three unique properties: self-renewal ability [1], differentiation capability [2], and exceptional proliferation potential [3, 4]. Due to their intrinsic ability to self-renew and differentiate into multiple lineages [2, 3], stem cells have been applied to generate complex tissues and organoids [5–9]. However, stem cells also have the capacity to develop tumors through cellular growth, recurrence, and metastasis [10]. Therefore, some of stem cells, which possess not only stem ability but also cancer ability, are classified as cancer stem cells (CSCs) [11]. CSCs can self-renew and differentiate into tumors, contributing to the growth, metastasis, and drug resistance of cancers. To understand the framework of these stem cells, including CSCs, several signaling pathways have been studied: Wnt signaling [12], Hedgehog signaling [13], Janus kinase/signal transducer and activator of transcription protein (JAK/STAT) signaling [14], Notch signaling [15], and the mammalian target of rapamycin (mTOR) signaling [16].

During the cellular proliferation of stem cells, the expression of Wnt protein and Hedgehog protein is upregulated [17]. Therefore, Wnt and Hedgehog signaling actively progress in stem cells, including CSCs, for self-renewal and proliferation [13]. In CSCs, JAK/STAT signaling pathway has been known to be associated with maintenance of self-renewal property, hematopoiesis, and neurogenesis [14, 18]. After phosphorylation, both JAK and STAT proteins are dimerize, leading to the activation of downstream transcription. As the Notch protein acts as a promoter or suppressor during transcriptional process, Notch signaling is considered significant in the regulation of CSCs’ properties, including differentiation into various types of tumor cells [15]. The Notch pathway’s effect depends on the types of substances binding to the receptors.

**Fig. 1:**
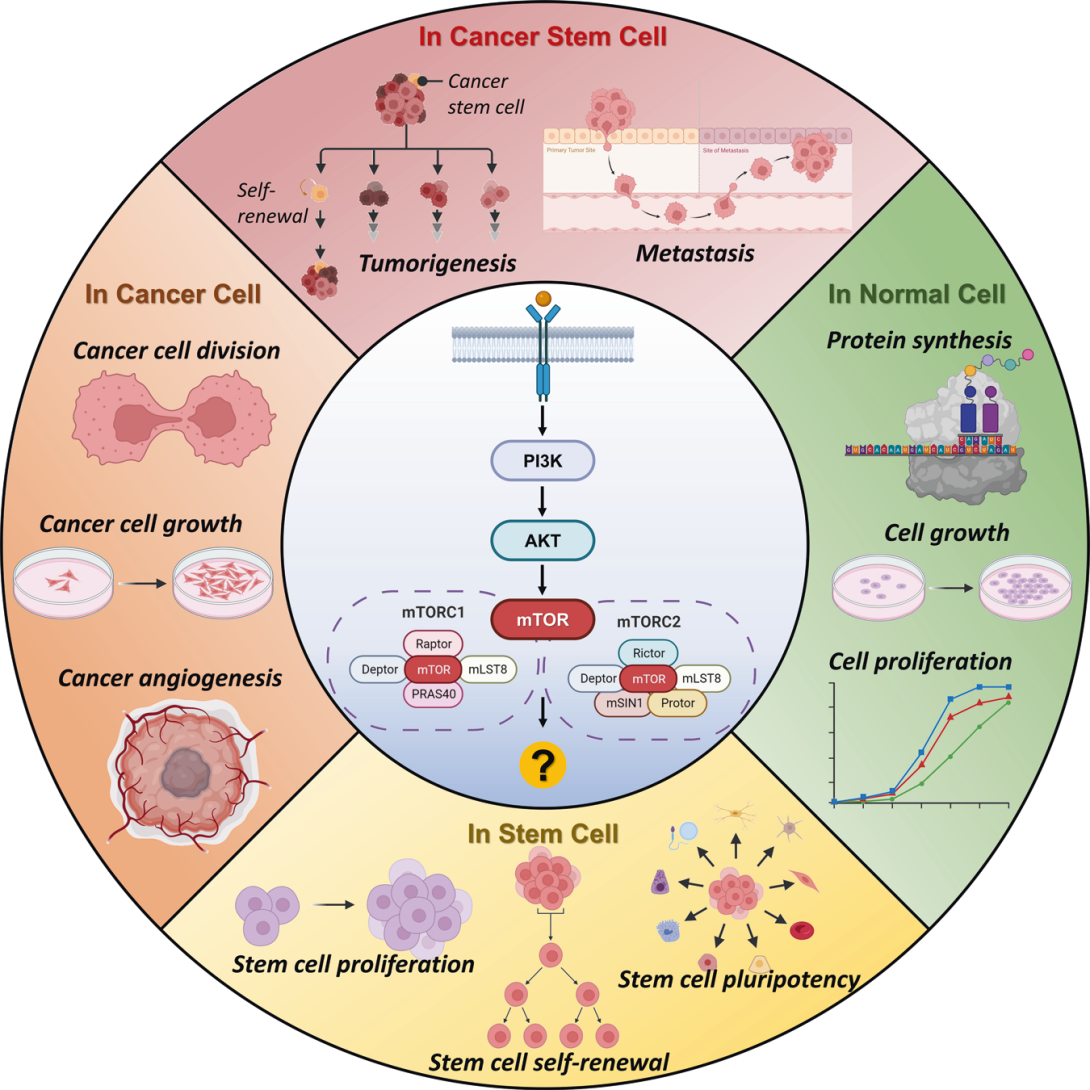
The mTOR signaling pathway in normal cells, stem cells, cancer cells, and CSCs. Effect of PI3K/AKT/mTOR axis is described depending upon types of cells. Normal cells can synthesize proteins, grow, and proliferate through activation of mTOR pathway [38, 41]. Stem cells can proliferate and self-renew through mTOR signaling [11, 30–32]. Also, pluripotency of the stem cells can be maintained by mTOR axis [33–35]. In cancer cells, cell division resulting in cell growth and angiogenesis are affected by mTOR signaling pathway [39, 40]. In CSCs, which have both characteristics of stem cells and cancer cells, unlimited tumorigenesis and metastasis are enhanced due to improved cell proliferation and angiogenesis [19–23].

In addition to those signaling pathways, mTOR signaling has been considered the most important for understanding and regulating CSCs [19–23]. mTOR, also referred to as FK506-binding protein 12-rapamycin-associated protein 1 (FRAP1), belongs to the phosphatidylinositol 3-kinase (PI3K)-related protein kinase family and is a component of both mTOR complex 1 (mTORC1) and mTOR complex 2 (mTORC2) [24–27]. mTOR is a serine/threonine kinase that regulates the cell cycle [28], glycolysis [28], the immune response [29], and many other activities in mammalian cells. Particularly, mTOR signaling is known to be essential for regulating the self-renewal ability, differentiation capacity, and proliferation property of the stem cells [11, 30–32]. It has been revealed that mTOR maintains pluripotency in human embryonic stem cells (hESCs) by preserving the expression of pluripotency-related genes such as *octamer-binding transcription factor 4* (*OCT4*), *NANOG*, and *sex determining region Y-box 2* (*SOX2*) [33, 34]. This cascade is related to upstream signaling pathway such as the PI3K/protein kinase B (AKT) pathway in pluripotency maintenance [35]. However, in mesenchymal stem cells (MSCs), in contrast to hESCs, mTOR signaling induces differentiation into various types of cells, including myoblasts, osteoblasts, adipocytes, and neurons [36]. In hematopoietic stem cells (HSCs), hyperproliferation and loss of quiescence occur due to inhibition of the mTOR pathway [37]. Therefore, the mTOR pathway is concluded to have different cellular effects depending on the types of stem cells. The mTOR signaling pathway not only in stem cells but also in various types of cells such as normal cells, cancer cells, and CSCs is described in Fig. 1.

Since cancers originate from CSCs, and mTOR signaling plays an important role in CSC regulation, the clinical potential and importance of mTOR in effective therapy are increasing [38]. Through anti-cancer therapy targeting CSCs by precisely regulating the mTOR pathway, the effectiveness of chemotherapy could be enhanced, potentially eliminating cancer metastasis and recurrence [39, 40]. In this review, we demonstrate the association between the mTOR pathway and cancers, including CSCs. Furthermore, we discuss how to develop new anti-cancer drugs and overcome the drug resistance, which is known to be the most challenging hurdle in CSC-targeting treatments.

## Cancer and mTOR pathway

Since mTOR signaling is strongly linked with CSCs, it is also associated with cancer, which typically originates from CSCs [41]. mTOR in cancer is related to tumorigenesis [42, 43], metastasis [44, 45], tumor development [46, 47] and angiogenesis [48, 49]. The mTORC1/ribosomal S6 kinase (S6K) pathway contributes to growth signal-mediated genome instability and tumorigenesis by inhibiting of the function of ring finger protein 168 (RNF168) [50]. Additionally, mTORC2/AKT signaling activates proliferative cell cycles in cancer cells, leading to tumor development, through the binding of mutated rat sarcoma virus (RAS) proteins to mTOR components of mTORC2 and mitogen-activated protein kinase-associated protein 1 (MAPKAP1) [51].

**Fig. 2:**
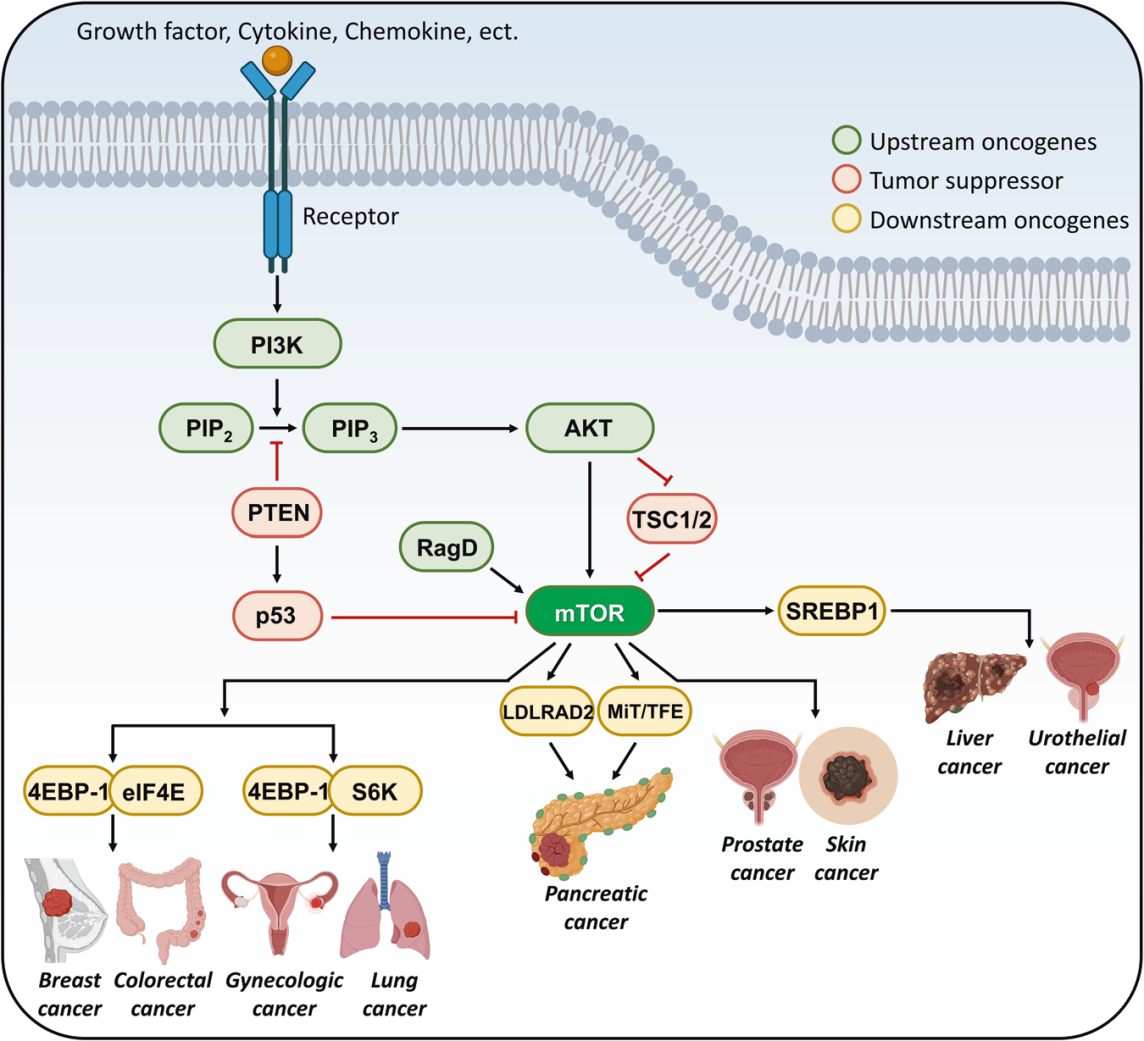
The mTOR pathway contributing tumorigenesis and tumor development. The PI3K/AKT/mTOR axis and related molecules, classified as oncogenes (green and yellow) and tumor suppressor (red), are described depending upon types of cancer. PTEN and p53 are the representative tumor suppressor [60–66, 112, 137, 138], whereas 4EBP-1 and SREBP1 are major downstream oncogenes. Breast and colorectal cancer are affected by 4EBP-1 and eIF4E [59–76], and gynecologic and lung cancer are induced by 4EBP-1 and S6K [77–83, 102–104]. Both LDLRAD2 and MiT/TFE are significant and direct oncogenes causing pancreatic cancer [193–196]. Liver and urothelial cancer are associated with expression of SREBP1 [180–184].

Abnormal activation of mTOR pathway leads to anabolism and energy storage, supplying a plethora of nutrients to the tumor, thus promoting tumor proliferation. Furthermore, mTOR regulates the expression of survival factors in cancer cells, such as cellular myelocytomatosis (C-MYC), and hypoxia induced gene 1 (HIG1), as well as angiogenic factors, including vascular endothelial growth factor (VEGF) [52–55]. Consequently, activation of the mTOR pathway improves and accelerates the generation, metastasis, proliferation, and angiogenesis of tumor cells [56, 57]. In Fig. 2, molecules contributing to mTOR pathway-mediated tumorigenesis and tumor development are described according to the types of cancer.

### Breast cancer

Breast cancer, a malignant tumor that originates in mammary glands, has been a significant cause of cancer-associated death, not only in women but also in men [58]. In breast cancer cells, the PI3K/AKT/mTORC1/sterol regulation element-binding protein (SREBP) pathway is recognized as the primary cascade inducing lipid synthesis and increasing the proliferation of tumor cells [59]. The tumor suppressor gene, phosphatase and tensin homolog (PTEN), plays an important role in breast cancer [60, 61]. Since PTEN regulates the PI3K/AKT/mTOR signaling pathway, it is involved in cell growth, survival, migration, and progression [62, 63]. Mutation in the phosphatidylinositol-4,5-bisphosphate 3-kinase catalytic subunit alpha (PIK3CA) gene and alterations in the breast cancer type 1 and 2 susceptibility protein (BRCA 1 and 2) genes lead to PTEN inactivation, resulting in the overexpression of the human epidermal growth factor receptor 2 (HER2) gene and the activation of the PI3K pathway [64–66]. It is known that 18 ~ 20% of breast cancers are caused by the overexpression of HER2 oncogenes, which activate the PI3K/AKT/mTOR pathway, promoting tumor development [65].

### Colorectal cancer

Colorectal cancer, including colon cancer, consists of malignant tumor cells in the large intestine tissues [67, 68]. In colorectal cancer, downstream molecules of the mTOR pathway such as eukaryotic translation initiation factor 4E (eIF4E) and eukaryotic translation initiation factor 4E-binding protein 1 (4E-BP1), play a crucial role in cancer development [69–72]. In colon cancer cell lines with overexpressed eIF4E, uncontrollable cellular growth and transformation into malignant forms were observed [73, 74]. In a transgenic in vivo mouse model induced to overexpress eIF4E, ubiquitination of the β-actin promoter was induced, and therefore, the development of tumor was also investigated [75]. Additionally, activated 4E-BP1, which was highly expressed in colon carcinoma, was found to be important for lymph node metastasis in colorectal cancer patients [76].

### Gynecologic cancer

Gynecologic cancer occurs in women’s reproductive organs and includes endometrial cancer (EC), cervical cancer (CC), ovarian cancer (OC), and others [77, 78]. In Phase II clinical trial, mTOR inhibition showed a significant effect on EC treatment; however, severe side effects such as diarrhea were also observed [79]. Other clinical trials have been conducted targeting the mTOR signal to explore its correlation with EC development [80, 81]. In the case of CC, blocking the phosphorylation of 4E-BP1 in human papillomaviruses (HPVs)-infected cells led to an increased number of cells in the G1 phase, resulting in apoptosis of the CC cells [82]. When mTOR inhibitor was applied solely to OC cells, it exhibited a moderate response, whereas dual inhibitor showed effective inhibition of the PI3K signaling pathway, deactivating the proliferation of the OC cell line [83].

### Liver cancer

When hepatocytes are damaged due to various external factors such as viruses, alcohol, and obesity, diseases are caused and then maintained chronically. The repetition of cell death and regeneration during disease symptoms eventually leads to the generation of hepatocellular carcinoma (HCC) [84]. The major causes of failure in liver cancer treatment are the invasion and metastasis of hepatocellular carcinoma (HCC) [85]. Since the PI3K/AKT/mTOR pathway is activated in HCC, activated mTOR serves as a representative marker for detecting the recovery or recurrence of liver cancer, including cholangiocarcinoma [86, 87] and hepatoblastoma [88, 89]. Therefore, expression of phosphorylated mTOR (p-mTOR) signifies and increased tumor grade, and elevated alpha-fetoprotein (AFP) indicates enhanced metastasis [90, 91], invasion [92, 93], and proliferation [94] of liver cancer cells.

Tumor cells invaded and metastasized into other tissues by degrading the extracellular matrix (ECM) and the basement membrane. Matrix metalloproteinase (MMP), the ECM-degrading enzyme, has been considered crucial for tumor invasion and metastasis. Among MMPs, MMP-2 and -9 have been highlighted as key factors in this function [95, 96]. The PI3K/AKT/mTOR pathway upregulates the expression of both MMP-2 and -9, thus promoting the invasion and metastasis of HCC [97, 98]. mTORC2 is also associated with liver cancer, as it induces the synthesis of fatty acids and lipids. The mTORC2 pathway has been a significant target for eliminating lipid-related liver tumors [99, 100]. According to the results from in vivo liver cancer mouse model, hepatic steatosis and the development of HCC were observed through the activation of the mTOR signal [101].

### Lung cancer

Lung cancer has two subtypes: non-small cell lung cancer (NSCLC) and small cell lung cancer (SCLC). Most cases of lung cancer are NSCLC, with only about 15% being SCLC [102]. Recently, it has been revealed that the PI3K/AKT/mTOR signaling pathway influences the aggressiveness of lung cancer. It stimulates transcription factors, cytokines, and receptor tyrosine kinases (RTKs), activating epithelial-mesenchymal transition (EMT) markers, which in turn cause invasion and migration in lung cancer [103]. Since the mTOR pathway is upregulated in NSCLC, the concentration of p-mTOR increases by up to 90% in NSCLC patients with adenocarcinoma, and up to 60% and 40% in NSCLC patients with large cell carcinoma and squamous cell carcinoma (SCC), respectively [103]. Additionally, the concentration of representative downstream molecules of the mTOR signal, such as ribosomal protein S6 (rpS6) and 4E-BP1, increased by up to 58% in NSCLC patients [103, 104].

### Pancreatic cancer

Pancreatic cancer is one of the leading causes of cancer-related deaths worldwide [105, 106]. It is known that low-density lipoprotein receptor class A domain-containing 2 (LDLRAD2) plays a significant role in the progression of pancreatic cancer [107–109]. LDLRAD2 is a gene involved in various human diseases, particularly common in pancreatic cancer cell lines and tissues. While LDLRAD2 is primarily regulated by the Wnt/β-catenin pathway, it has been also found that phosphorylation of AKT and mTOR molecules is significantly reduced in an LDLRAD2 knockout model [110]. Knocking out LDLRAD2 successfully decreases the proliferation, invasion, and metastasis of pancreatic cancer cells [110]. Therefore, it has been confirmed that LDLRAD2 is involved in the progression of pancreatic cancer by regulating the AKT/mTOR pathway. Additionally, RAS-related guanosine triphosphate (GTP)-binding protein D (RRAGD)-mediated activation of mTORC1 has been observed in pancreatic ductal adenocarcinoma patients, and an increase in proliferation and cancer growth has also been investigated [111].

### Prostate cancer

Prostate cancer is the second leading cause of cancer-related death in men, following lung cancer [112]. In prostate cancer, mTOR downregulates the expression of glycogen synthase kinase 3 (GSK-3) [113]. The decrease in GSK-3 inhibits the caspase-3 signaling pathway, reducing ROS production. Consequently, the apoptosis of tumor cells is inhibited [113]. Thus, mTOR activation induces the development of prostate cancer by inhibiting cellular apoptosis.

### Skin cancer

Skin cancer is divided into various types, represented as melanoma and keratinocyte carcinoma, depending on the area it occurs and its relationship to the mTOR pathway [114, 115]. In melanoma patients, the level of phosphorylated AKT (p-AKT) level is high, and the PI3K/AKT/mTOR pathway is activated due to PTEN loss [116]. It has already been found that PTEN is reduced by 10 ~ 30% in melanoma cell lines [117]. Serine/threonine protein kinase (BRAF), a member of the RAF kinase family, plays a vital role in regulating the MAPK pathway, influencing cell division, differentiation, and secretion [118–120]. Mutation of BRAF is known to induce melanoma genesis and metastasis [117]. The BRAF mutation is found in 60% of melanoma cells [121] and 90% of melanoma patients [122].

In keratinocyte carcinoma, there are three major types of cells: basal cell carcinoma (BCC), SCC, and Merkel cell carcinoma (MCC). The generation of BCC is strongly linked to the PI3K/AKT/mTOR pathway [123]. Treatment of an mTOR inhibitor showed significant disease recession in BCC patients [124]. SCC exhibits a higher mTOR level than BCC [125, 126]. Furthermore, enhanced expression of cyclic-dependent kinase 2 (CDK2) induces the malignant transition of cancer cells through AKT/mTOR signal [127]. MCC, a nonmelanoma skin cancer derived from neuroendocrine cells, is associated with the activation of 4E-BP1 and rpS6, downstream molecules of the mTOR pathway [128]. Administering a dual drug that inhibits both mTORC1 and mTORC2 in a xenograft mouse model effectively suppressed the growth of MCC [129, 130].

### Urothelial cancer

Urothelial cancer is a type of tumor that can occur in the urethra, bladder, ureters, renal pelvis, and other urinary organs [131]. Bladder cancer is the fifth most common cancer in the world and the most common cancer among urothelial cancers [132]. In bladder cancer, the AKT/mTOR signaling pathway is activated through the upregulation of pyruvate kinase M2 (PKM2), leading to increased expression of SREBP-1c and the genes related to fatty acid synthesis [133, 134]. As fatty acid production increases, tumor growth is also enhanced [51]. PTEN, a suppressor of the mTOR signal, is related to various types of urothelial cancers and their mechanisms [135, 136]. In particular, the p53 pathway, one of the PTEN downstream pathways, has the greatest influence on bladder cancer, inducing apoptosis in tumor cells [132, 137]. Therefore, inactivation of PTEN and p53 together increases mTOR signaling and promotes tumor growth [132, 138]. Phosphorylated-rpS6 (p-rpS6), as a marker of mTOR activity, is frequently expressed in muscle-invasive urothelial cancer cells [139–141]. Thus, p-rpS6 is associated with lymph node metastasis. According to related studies, it was demonstrated that cell proliferation and migration decreased, and the tumor volume was also reduced by 55% in the T24-xenograft in vivo model through the inhibition of rpS6 phosphorylation and subsequent inactivation of the mTOR pathway [142].

## Targeting mTOR signaling In Cscs

Cancer cells are not composed of only one type of cell but are a group of multiple cells [143, 144]. This characteristic is called heterogeneity, and research on various types of cancer has been ongoing since the 1800s [76, 145]. Therefore, tumor cells exhibit diverse characteristics and functions that support cancer development [146].

Initially, CSCs were identified in leukemia in 1994 as a type of cancer cell with the ability to continuously grow and self-renew [147]. It was revealed that a small subset of cancer cells could (re)generate tumor cells, leading to investigations into the capacity of self-renewal and differentiation into heterogeneous tumor cells [148]. There have been various opinions and contentions regarding the crucial role of CSCs, which represent only a small portion of the total cancer cells within a tumor that contains various cell types.

CSCs are believed to be generated within a niche, which includes fibroblasts, blood vessels, and secreted factors that surround and influence CSCs [149]. This process is similar to normal stem cells, and the accumulation of many mutations in stem cells has been considered an important factor in their conversion into CSCs [150, 151]. Another hypothesis suggests that cancer cells can mutate into CSCs during the chemotherapy process of metastasis [152]. Metastatic cancer cells can acquire the ability to differentiate into other types of cancer cells, resulting in the generation of CSCs [150, 153]. According to another viewpoint, when a small subset of cancer cells is stimulated and activated by chemotherapy, they can differentiate into cancer cells with the ability to induce cancer recurrence [154, 155].

**Fig. 3:**
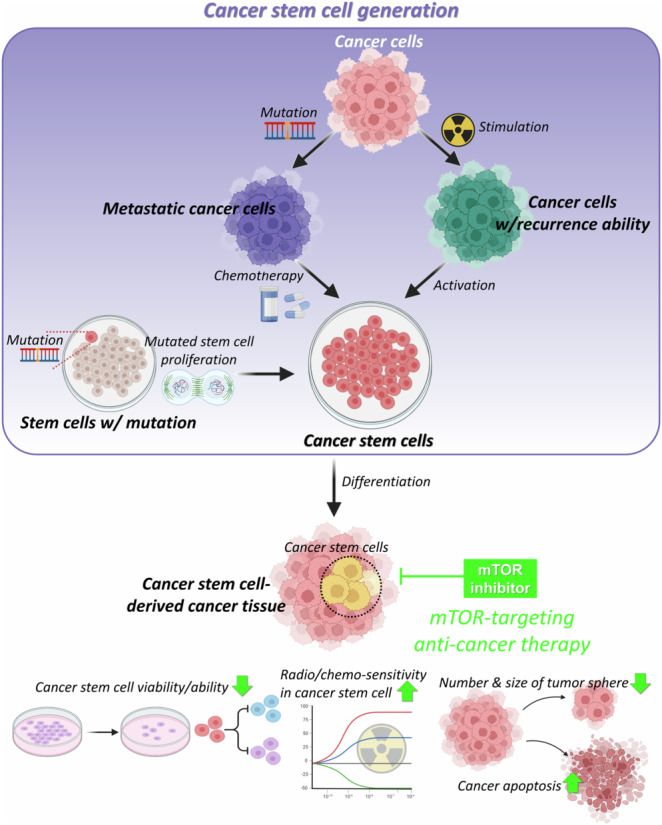
Hypothesis on CSC generation, and CSC-targeting therapy through inhibition of mTOR pathway. There have been several hypotheses on how the CSCs, which possess a crucial role in cancer development, are generated (purple box in left). Accumulation of mutations in normal stem cells are considered as an important cause of CSC generation [150, 151]. Also, cancer cells can be mutated into CSCs by chemotherapy during the process of metastasis [152]. The metastatic cancer cells can acquire the ability to differentiate into other types of cancer cells, resulting in generation of CSCs [150, 153]. In addition, when a small part of the cancer cells is stimulated and activated by radiotherapy and chemotherapy, it can be differentiated into cancer cells, which have ability to induce cancer recurrence [154]. And these cancer cells with recurrence ability can be activated into CSCs [155]. Through differentiation of the CSCs, CSC-derived cancer tissues are generated (middle of the scheme). Through treatment of mTOR inhibitor to the CSC-derived cancer tissues, CSC-targeting anti-cancer therapy is possible (pink box in right). By inhibition of mTOR signaling pathway, viability and ability of CSC decrease [89, 95, 97–99, 102, 109], radio/chemo-sensitivity of the CSCs increase [100], and number or size of tumor sphere decrease [87, 88, 90–92, 108, 111], resulting in apoptosis of the cancer tissues [86, 96, 112, 115].

Figure 3 depicts the hypothesis on CSC generation, followed by the results of CSC-targeting anti-cancer therapeutics through mTOR inhibition. Additionally, various types of mTOR inhibitors and their outcomes are presented, depending on the types of CSCs, in Table 1.

**Table 1:** Pre-clinical research targeting cancer stem cells with mTOR inhibitors in vitro depending upon types of cancers.

Cancer type	Drug name	Target molecules	Co-treatment	Results	Refs
Bone marrow cancer stem cell	S14161	PI3K, AKT, mTOR	-	Reduction in proportion of SP cells	[113]
Breast cancer stem cell	Metformin	mTOR, AMPK	-	Reduction in number and size of tumor spheres	[87, 88]
			FEC	Decrease of CSC ability	[89]
	Rapamycin	mTORC1	-	Reduction in number of mammospheres Increase in radio-sensitivity of CSCs	[90]
	VS-5584	mTORC1/2, PI3K	-	Reduction in number of tumor spheres Induction of CSC apoptosis	[86]
			Paclitaxel	Reduction of CSC population	
CNS cancer stem cell	Alpelisib	PI3K, AKT, mTOR	-	Increase in anti-neoplastic effects of CSCs	[103]
	Dactolisib	PI3K, mTOR	-	Reduction in tumorigenicity and proliferation of CSCs Increase in radio-sensitivity of CSCs	[101, 102]
	AZD2014	mTORC1/2	-	Increase in radio-sensitivity of CSCs	[100]
	FC85	AKT, mTOR	ISA27	Induction of CSC apoptosis Reduction of CSC viability Promotion of CSC differentiation	[96]
	Rapamycin	mTORC1	-	Reduction of CSC proliferation	[98, 99]
			ATRA		[97]
Colon cancer stem cell	Dactolisib	PI3K, mTOR	-	Reduction of CSC stemness and proliferation	[94]
				Increase of CSC differentiation	[93]
	PF-04691502	PI3K, mTOR	-	Reduction of CSC proliferation	[95]
	Rapamycin	mTORC1	-	Increase of CSC differentiation	[93]
			pp242	Reduction in tumor-sphere formation capacity of CSCs	[91, 92]
	Torin-1	mTORC2	-	Reduction in growth, motility, invasion and survival of CSCs	[91]
Liver cancer stem cell	Rapamycin	mTORC1	DAPT	Increase of CSC markers	[104]
	Temsirolimus	mTORC1	Baicalein	Elimination of dug resistance of CSCs	[105]
Lung cancer stem cell	β-elemene	PI3K, AKT, mTOR	Cisplatin	Induction of CSC apoptosis	[112]
	Rapamycin	mTORC1	-	Reduction in sphere formation of CSCs	[111]
Ovarian cancer stem cell	Dactolisib	PI3K, mTOR	Cisplatin	Reduction in colony formation ability, EMT, and marker expression of CSCs	[114]
Pancreas cancer stem cell	AZD8055	mTORC1/2	BMS-777607	Reduction of CSC viability	[109]
	Dactolisib	PI3K, mTOR	-	Reduction of tumor growth Decrease in self-renewal capacity, pluripotency factors, and EMT factors of CSCs	[106]
	Rapamycin	mTORC1	-	Reduction in sphere formation and anchorage-independent growth of CSCs	[107]
			GANT61	Reduction in sphere formation of CSCs	[108]
Salivary gland cancer stem cell	Rapamycin	mTORC1	-	Induction of CSC apoptosis	[115]
	Temsirolimus				
Smooth muscle cancer stem cell	Dactolisib	PI3K, mTOR	-	Reduction in stemness properties of CSCs	[110]

### Breast CSCs

Rapamycin (ABI-009), a representative mTORC1 inhibitor, has been approved by the U.S. Food and Drug Administration (FDA) as an immunosuppressant for renal transplantation and for the treatment of lymphangioleiomyomatosis (LAM) [156, 157]. Furthermore, rapamycin (ABI-009) is being considered for use as an anti-cancer drug targeting the mTOR signal in breast cancer stem cells [158]. Since rapamycin (ABI-009) inhibits the mTOR pathway, resulting in the downregulation of manganese superoxide dismutase (MnSOD) expression, it has been revealed that rapamycin (ABI-009) reduces the number of mammospheres, which are the markers of breast cancer development, and makes breast CSCs sensitive to radiation therapy [159].

Metformin is a drug that inhibits the mTOR pathway by activating adenosine monophosphate-activated protein kinase (AMPK) [160]. When metformin was treated to breast CSCs, the size and number of the CSCs significantly decreased, and their radiosensitivity increased [161, 162]. In addition, metformin reduced the recurrence ability of breast CSCs when co-treated with fluorouracil (5-FU), epirubicin, and cyclophosphamide (FEC) [163].

Investigations have also been conducted to assess the effects of another mTOR inhibitor, everolimus (RAD001) [99, 107, 111, 164, 165]. According to the results, everolimus (RAD001) was found to increase the expression of caspase-3 and -8 in breast cancer cells, inducing apoptosis of the tumors by upregulating apoptotic gene expression [99, 107]. Furthermore, it was confirmed that the growth of breast cancer cells decreased with everolimus (RAD001) treatment in an MCF-7 bearing in vivo mouse model [107]. Additionally, co-treatment of everolimus (RAD001) with the anti-estrogen drug exemestane (FCE-24304) for hormone receptor-positive breast cancer therapy showed alleviation of breast cancer by inactivating the mTOR signal in patients [111, 164, 165].

As a dual inhibitor targeting both PI3K and mTOR, VS-5584 (SB2343) efficiently decreased the number of tumor spheres and induced apoptosis of CSCs when co-treated with paclitaxel (PTX), which regulates the cell cycle to stay at G2, inhibiting the cell division [166, 167].

### Central nervous system CSCs

Glioblastoma multiforme (GBM) is one of the most aggressive cancers that occur in the central nervous system (CNS) and is known to be difficult to treat due to strong resistance to chemotherapy [168]. FC85, a drug targeting AKT/mTOR signal, reactivates the functionality of p53 by blocking its endogenous inhibitor, murine double minute 2 homologue (MDM2) [169]. Since p53 is a tumor suppressor that induces apoptosis in cancer cells, co-treatment of FC85 with ISA27 influences the promotion of differentiation and the inhibition of proliferation in GBM CSCs [169, 170].

All-trans retinoic acid (ATRA), derived from retinol, is known to induce the differentiation of neuro-related progenitor cells and stem cells [171]. Therefore, co-treatment of ATRA with rapamycin (ABI-009) reduces the size of tumor and decreases the motility of cancer cells, inducing differentiation of the CSCs in GBM [171].

In addition to GBM, neuroblastoma is another type of CNS cancer derived from neural crest tissue that frequently occurs in young people [172]. To investigate the anti-cancer efficiency of triciribine, an AKT inhibitor, co-treatment with rapamycin (ABI-009) was applied to both GBM cell line (U251) and the neuroblastoma cell line (SH-SY5Y) [173]. Inhibition of proliferation and reduction in migration were observed in both cell lines. Furthermore, rapamycin (ABI-009) was applied to CSCs derived from GBM and neuroblastoma, resulting in successful suppression of cell growth and downregulation of CD133, indicating the inhibition of the neural stem cell surface marker expression [174].

### Colon CSCs

In human metastatic colon cancers, including colon CSCs, mTORC2 is strongly expressed, and thus, the mTORC2 pathway activates serum/glucocorticoid-regulated kinase 1 (SGK1), enhancing the cancer properties [175]. Therefore, one of the most popular mTORC2 inhibitors, torin-1, has been used to reduce the growth, survival, and invasion of colon CSC populations [175]. Accordingly, torin-1 decreased the stem cell markers related to pluripotency in colon CSCs in an in vivo model.

Another mTOR inhibitor, torkinib (PP242), was also treated with rapamycin (ABI-009), resulting in reduced tumor formation ability and aldehyde dehydrogenase 1 (ALDH1) activity, inducing autophagy [176]. Additionally, co-treatment of rapamycin (ABI-009) with dactolisib (NVP-BEZ235) caused the colon CSCs to lose their differentiation capability due to the dual inhibition of PI3K/mTOR [177]. It was confirmed that treatment of dactolisib (NVP-BEZ235) alone inhibited the proliferation of colon CSCs and reduced the expression of stem cell markers such as CD133 and leucine-rich repeat-containing G-protein coupled receptor 5 (LGR5) [178].

As an ATP-competitive PI3K/mTOR dual inhibitor, gedatolisib (PF-04691502) has been utilized to inhibit tumor growth and CSC proliferation in both in vitro and in vivo colon cancer models [179].

### Liver CSCs

Since HCC causes mutations in hepatic progenitor cells, liver CSCs are generated and activated, resulting in chemoresistance, tumor relapse, and metastasis [180]. Sorafenib and regorafenib (BAY 73-4506), the representative multi-kinase inhibitors used in patients with advanced HCC, lose their effectiveness due to drug resistance with frequent administration [181]. Therefore, to overcome resistance issues in liver CSCs, rapamycin (ABI-009) was administered before the sorafenib. This resulted in the suppression of mTOR by rapamycin (ABI-009) and a reduction in the liver CSC population by sorafenib. Additionally, treatment with sorafenib followed by rapamycin (ABI-009) decreased the proportion of liver CSCs and reduced their ability to form tumor spheres [182].

Baicalein, a drug used to regulate inflammatory factors and treat cancer [183], was co-administered with temsirolimus (CCI-779) to an in vivo animal model. This combination prevented HCC growth and also suppressed the ability of autophagy [184].

### Lung CSCs

Recently, studies have shown that CSCs are initiated during tumor development and affect tumor progression and metastasis in lung cancer [185, 186]. In lung CSCs, overexpression of CD164 and activation of AKT/mTOR signaling pathway have been observed [187, 188]. Therefore, rapamycin (ABI-009) has shown significant effects in pre-clinical investigations by suppressing sphere formation and tumor growth. This is achieved through the upregulation of C-X-C motif chemokine receptor 4 (CXCR4), which is involved in the growth and metastasis of lung cancer cells [187].

Cisplatin (CDDP), an anti-cancer drug most effective against tumor cells in the resting period, and β- elemene, which inhibits the PI3K/AKT/mTOR axis, were co-treated to lung cancer cell lines such as A549 and NCI-H1650 [189]. According to the results, the combination treatment reduced resistance to chemotherapy and stem-like behaviors. In clinical trials, rapamycin (ABI-009) was also investigated in a phase I clinical trial through co-treatment with sunitinib (SU11248), which targets receptors for NSCLC cell growth [190]. Additionally, temsirolimus (CCI-779), an mTORC1 inhibitor, was studied in a phase II clinical trial for patients with stage III NSCLC or stage IV NSCLC [191]. Furthermore, the dual mTOR inhibitor, sapanisertib (MLN0128), was tested in a phase II clinical trial for patients with lung cancer, which is at stage IV or recurrent due to mutation in multiple genes related to cancer growth [192].

### Pancreatic CSCs

In the case of pancreatic cancer, CSCs frequently contribute to cancer relapse and induce resistance to typical therapeutics [193]. Co-administration of sonidegib (LDE225), a PI3K/mTOR inhibitor, with dactolisib (NVP-BEZ235), a smoothened inhibitor, in an in vivo mouse model, resulted in the downregulation of pluripotency markers such as OCT4, NANOG, SOX2, and c-MYC in pancreatic CSCs. This led to suppression of cancer growth and the formation of tumor spheres [193]. Additionally, the combination treatment of sonidegib (LDE225) with dactolisib (NVP-BEZ235) regulated genetic expression related to cellular proliferation and apoptosis [193].

Rapamycin (ABI-009) is known to inhibit formation of tumor spheres and anchorage-independent growth in pancreatic cancer cells [21]. Therefore, when rapamycin (ABI-009) was co-administered with glioma-associated inhibitor 61 (GANT-61), a Hedgehog signal inhibitor targeting Zinc finger protein GLI1, a reduction in cell viability and sphere growth was observed [194].

BMS-777607, a small-molecule met kinase inhibitor, also inhibits the tyrosine kinase of the MET receptor and is known to have therapeutic effects in tumor xenograft in vivo model [195]. Thus, BMS-777607 was co-treated with an mTOR inhibitor AZD8055, to pancreatic CSCs. The combination treatment not only reduced cell viability but also enhanced therapeutic synergy effects [196].

### Other CSCs

Experiments involving various drugs have also been conducted on several other CSCs. For example, dactolisib (NVP-BEZ235), a dual inhibitor targeting both PI3K and mTOR, was co-treated with cisplatin (CDDP) to ovarian CSCs, leading to a reduction in stem cell colony formation and downregulation of stem cell markers [197]. Additionally, treatment with dactolisib (NVP-BEZ235) reduced stem cell properties in smooth muscle CSCs [198].

Another trial-targeting drug that inhibits three molecules (PI3K, AKT, and mTOR), S14161, has been found to significantly reduce the proportion of side population cells (SP cells) with stem cell-like characteristics [199].

To inhibit mTOR of mTORC1, rapamycin (ABI-009) was co-treated with temsirolimus (CCI-779) to salivary grand CSCs, resulting in promotion of apoptosis in the cells [200]. Rapamycin (ABI-009), being one of the most popular and effective mTOR inhibitors, was also applied to nasopharyngeal CSCs, which occur in the throat, nose, and back of the mouth [201].

Consequently, when drugs inhibiting mTOR signaling were used in diverse combinations on various types of CSCs, mostly the mTOR inhibitor showed a significant effect on reducing stem cell markers and cellular proliferation, leading to a decrease in tumor development.

## Efficient targeting Of Cscs

CSCs have been considered as ‘cancer-initiating cells’, performing a pivotal role in tumor relapse, metastasis, and resistance to radiotherapy and chemotherapy [202, 203]. Tumors often regrow after treatments because the regrowth process is accelerated, and the self-renewal ability of CSCs remains unrestricted. Hence, there is a need for CSC-targeting therapy to prevent tumor recurrence [204–207].

### Targeting CSC biomarkers

There have been some representative biomarkers which have been used to identify and efficiently target the CSCs [208, 209]: CD44, CD133, epithelial cell adhesion molecule (EpCAM), and ALDH. CD44, a receptor for hyaluronic acid (HA) in ECM, promotes anti-apoptosis and chemo-resistance in CSCs by activating signaling pathways such as STAT3 and c-Src kinase [210–212]. CD133, another well-characterized biomarker, is associated with tumorigenicity, spheroid formation, and EMT [213–215]. Since EpCAM regulates cell adhesion, proliferation, migration, and EMT, overexpression of EpCAM is directly linked to tumor formation and poor differentiation grade [216–218]. ALDHs, crucial in aldehyde metabolism, are upregulated in highly malignant tumors and contribute to CSC maintenance through interactions with retinoic acid, reactive oxygen species (ROS), and chemotherapeutic drugs [219–222]. SOX2/OCT4 and Wnt/β-catenin signaling pathways interact with ALDH to maintain CSC stemness and therapy resistance [223–225]. Although those identified CSC markers have been used to target the CSCs with high selectivity, not all of the CSC biomarkers are equally effective. Thus, profound research on efficacy comparison in vivo and multiple targeting is required for enhanced CSC-targeting.

Based on those studies, CSC biomarker-targeting agents have been investigated in some clinical trials [226]. Rituximab, targeting CD20, has shown efficacy in treating follicular lymphoma and mantle-cell lymphoma, although adverse reactions have been noted [227, 228]. Alemtuzumab, directed against CD52, has received approval for chronic lymphocytic leukemia (CLL) treatment, and combining CD20 and CD52 antibodies for refractory CLL has shown positive results [229]. For head and neck squamous cell carcinoma (SCC) treatment, bivatuzumab, targeting CD44v6, demonstrated safety [230, 231]. Further, various CD123 antibodies, such as XmAb14045, MGD006, and talacotuzumab, have been developed with improved effects, showing promise in clinical trials [232–234]. Additionally, research is also ongoing using EpCAM antibodies, like adecatumumab and catumaxomab, which have shown potential in treating hormone-resistant prostate cancer and ovarian cancer [235–238]. Also, combinational approaches involving EpCAM antibodies and CAR-T technology have been explored in phase II clinical trials for various cancers (NCT02729493, and NCT02725125).

Given the diversity in CSC surface markers of various types of cancers, developing effective treatment strategies requires a tailored approach. Each type of cancer may exhibit unique characteristics in terms of CSC markers, influencing how therapies are designed and conducted. Consequently, clinical trials must adopt combined approaches that account for these variations to maximize treatment efficacy. This approach ensures that treatments are specifically targeted to the CSCs present in each cancer type, thereby improving the chances of successful outcomes for patients undergoing experimental therapies.

### Targeting CSC pathways

Another CSC-targeting technology is based on CSC-associated key signaling pathways [226]: Notch [239, 240], Hedgehog [241], Wnt [242], TGF-β [243, 244], JAK-STAT [245, 246], PI3K [20], and NFκB [247]. Abnormal function of those signaling pathways is crucial in CSCs [226]. And these pathways often interact with each other through crosstalk during tumor development. Although Notch signaling, vital for CSC maintenance and differentiation, is dysregulated in various cancers, trials with Notch inhibitors showed mixed results across different cancers [248, 249]. For instance, a representative γ-secretase inhibitor (GSI) MK-0752, despite initial promise in leukemia, yielded poor outcomes in solid tumors [250, 251]. In case of Hedgehog signaling, which contributes to CSC stemness and chemoresistance [252–254], approved inhibitors like vismodegib, sonidegib, and glasdegib demonstrated efficacy in basal cell carcinoma and acute myeloid leukemia (AML) [255–257]. Trials combining these kinds of inhibitors with chemotherapy are ongoing for treatment of various cancers [257]. By targeting these CSC-associated pathways, CSC-driven tumor growth and treatment resistance could be relieved.

Thus, the integration of CSC-targeting strategies with mTOR-targeted therapeutics as outlined above holds promise for developing a novel therapeutic paradigm aimed specifically at mTOR inhibition in CSCs. This approach is designed to enhance the eradication of CSCs, potentially decreasing drug resistance mechanisms and preventing tumor recurrence. By selectively targeting mTOR in CSCs, this therapeutic strategy offers an advanced approach to anti-cancer treatment, aiming to achieve efficacy while minimizing off-target effects on non-CSC populations. Therefore, such a precise approach in targeting CSCs through mTOR inhibition emphasizes its potential as a safer and more effective treatment method. And further investigations in clinical settings to validate its therapeutic utility in cancer management are required.

## Recommendations for following research

### Combinational approaches with mTOR inhibition

Unlike other stem cells, CSCs have a considerably longer cell cycle due to the activation of anti-apoptotic signals, such as B cell lymphoma 2 (BCL-2) [258], and the induction of myeloid leukemia cell differentiation protein 1 (MCL-1) [259]. CSCs also possess a skillful DNA repair system, which significantly contributes to therapy resistance [260–262]. Furthermore, there are intrinsic factors involved in the development of resistance, such as oxidative modulators, metabolic plasticity regulators, and drug efflux-regulating pumps [263]. Among a variety of signaling pathways associated with the CSCs, PI3K/AKT/mTOR axis is deeply involved in cascades such as anti-oxidative mechanisms [264] and anti-quiescence signaling [265], resulting in cancer resistance and survival in the end. Consequently, numerous clinical trials have been conducted, involving various types of cancers treated with drugs that inhibit the mTOR pathway (Table 2). These trials have addressed breast cancer, colon cancer, lung cancer, ovarian cancer, pancreatic cancer, prostate cancer, and urothelial cancer, utilizing various mTOR inhibitors such as rapamycin, everolimus, metformin, and sirolimus. Since these sole treatments utilizing mTOR pathway inhibitors only have been faced with limitations especially in efficacy, there have been combinational approaches for enhanced anti-cancer therapy.

**Table 2:** Clinical trials for anti-cancer therapy using mTOR inhibitors depending upon types of cancers.

Cancer type	Drug name	Target molecules	Co-treatment	Clinical phase	Trial number
			Capecitabine	Phase1	NCT00473005
			Erlotinib	Phase1	NCT00574366
	Everolimus	mTOR	Lapatinib	Phase2	NCT01272141
			Tamoxifen	Phase2	NCT01298713
Breast cancer			Vinorelbine	Phase2	NCT01520103
	PF-04691502	PI3K/mTOR	Exemestane	Phase2	NCT01658176
	Ridaforolimus	mTOR	Trastuzumab	Phase2	-
	TAK-228	mTOR	Letrozole	Phase1	NCT02619669
			Tamoxifen	Phase2	NCT02988986
	XL765	PI3K/mTOR	Letrozole	Phase2	NCT01082068
Colon cancer	Everolimus	mTOR	Cetuximab	Phase2	NCT00522665
			Irinotecan	Phase1	NCT00522665
	Rapamycin	mTORC1	-	Phase2	NCT00409994
Lung cancer	CC-223	mTOR	Erlotinib and oral azacitidine	Phase1	NCT01545947
	Everolimus	mTOR	-	Phase1	NCT00401778
	Metformin	mTOR	-	Phase2	NCT02019979
	Sirolimus	mTOR	AMG954 and Panitumumab	Phase1	NCT00352950
			Gold sodium thiomalate	Phase1	NCT01383668
	Temsirolimus	mTOR	-	Phase2	NCT00079235
Ovarian cancer	Metformin	mTOR	Carboplatin and paclitaxel	Phase1	NCT02312661
	Temsirolimus	mTOR	-	Phase2	NCT00926107
			Vorinostat	Phase1	NCT01174199
Pancreatic cancer	Dactolisib	mTOR	-	Phase2	-
	Everolimus	mTOR	Capecitabine	Phase1	NCT01077986
			Cetuximab	Phase2	NCT01658436
	Metformin	mTOR	-	Phase2	NCT01210911
Prostate cancer	AZD2014	mTOR	-	Phase1	NCT02064608
	Everolimus	mTOR	Radiation therapy	Phase1	NCT01548807
	LY3023414	PI3K/mTOR	Enzalutamide	Phase2	NCT02407054
	MLN0128	mTOR	-	Phase2	NCT02091531
	Temsirolimus	mTOR	-	Phase1	NCT01020305
Urothelial cancer	Everolimus	mTOR	Imatinib mesylate	Phase2	NCT00331409
	Temsirolimus	mTOR	Nelfinavir	Phase1	NCT01079286

**Fig. 4:**
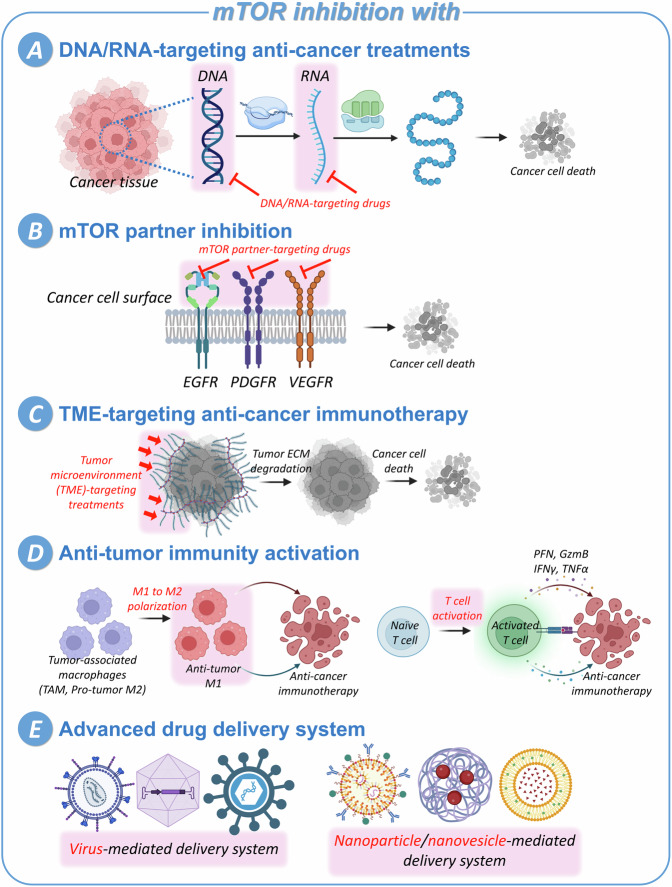
Advanced anti-cancer strategies which can be combined with CSC mTOR-targeting therapies. For enhanced anti-cancer treatment, mTOR-targeting strategy can be combined with DNA/RNA-targeting anti-cancer therapies (**A**), mTOR partner inhibition (**B**), TME-targeting immunotherapy (**C**), anti-tumor immunity activation through M1 macrophage polarization or T cell activation (**D**), and advanced drug delivery system using virus, nanoparticles, or nanovesicles (**E**).

First, DNA/RNA-targeting anti-cancer drugs have been co-treated with mTOR pathway inhibitors (Fig. 4A). Azacitidine priorly targets RNA and induces cytotoxicity in G1 phase cells [266], carboplatin targets replicating DNA inhibiting transcription [267], and capecitabine for oral administration disrupts DNA and RNA synthesis inducing cellular apoptosis especially in metastatic cancer [268, 269]. Second, drugs targeting mTOR partners which crosstalk with mTOR signaling pathway in diverse cancers have been used combined with mTOR inhibitors (Fig. 4B). Cetuximab, monoclonal antibody of epidermal growth factor receptor (EGFR), blocks activation of EGFR cascades through ligand binding inhibition, hindering cell proliferation [270]. Enzalutamide which targets androgen pathway [271], crenolanib, the representative inhibitor targeting platelet-derived growth factor receptor (PDGFR) signaling pathway [272], and numerous vascular endothelial growth factor receptor (VEGFR) inhibitors [273] have been co-treated with mTOR inhibitors.

Despite the ongoing efforts in combinational therapies targeting DNA/RNA or mTOR partners, it is crucial to acknowledge that the clinical outcomes derived from these approaches are currently limited to phases 1 and 2 trials. These early stages primarily focus on assessing initial safety profiles, optimal dosing, and preliminary effectiveness. Therefore, it remains imperative to conduct subsequent phase 3 trials and long-term studies to establish the definitive efficacy and safety of these treatments. Only through rigorous evaluation across diverse patient populations, we can verify their potential impact and usefulness as standard therapies in the future.

### Tumor immune modulation through mTOR inhibition

In addition to the intrinsic factors, one of the most well-known key factors contributing to resistance capabilities of the CSCs is tumor microenvironment (TME) [274–276]. The TME determines the fate of tumor by interacting with molecules in the surrounding cancer tissue [277, 278]. In TME conditions, there is an abundance of M2 macrophages, which mediate immunosuppression. Consequently, the anti-inflammatory environment induced by these M2 macrophages contributes to therapeutics resistance [263, 279–281]. Hence, to develop improved anti-cancer therapy, there have been recent approaches target the TME developed by tumor-associated immune systems. These approaches involve removing nutrients or signals essential to the TME, creating different environments that induce cancer quiescence [282, 283]. Additionally, the TME itself can be eliminated using enzymes that degrade the components of the ECM within the TME [284, 285]. In recent studies on immunotherapy, tumor-associated macrophages (TAM), specifically M2 macrophages with pro-tumor activity, have been induced to polarize into anti-tumor macrophages, known as inflammatory M1 macrophages. This immune modulation aims to alter the immune composition and properties of cancer circumstances [286, 287].

Since mTOR has been known to modulate not only the cancer tissue itself but also those tumor immune systems, the role and importance of the mTOR in innate and adaptive immune responses is recently spotlighted [288]. First, the mTOR is known to regulate antigen-presenting cells (APCs), such as dendritic cells (DCs) [289]. As a representative mTOR inhibitor, rapamycin induces differentiation of the APC, and inhibits antigen uptake by DCs, modulating their antigen presentation ability [290]. Second, mTOR is also known to activate effector T cells [291], and to induce proliferation of regulatory T cells (Tregs) [292, 293]. In detail, rapamycin-based mTOR inhibition affects the T cell activation process, disrupting some part of the cell cycle, and also separates the activated T cells, which are ready to fight [294]. Simultaneously, rapamycin induces the Tregs to proliferate, and increases the population of forkhead box P3-positive (FOXP3 + ) T cells, which will be changed into major immune modulators [295, 296].

Therefore, based on the understanding of the relationship between cancer and the immune system, it is anticipated that anti-cancer immunotherapies could maximize the efficacy of cancer treatments. By applying anti-cancer immunotherapies targeting the TME, it is possible to efficiently disrupt the interaction between TME and CSCs (Fig. 4C). Furthermore, by polarizing the abundant pro-tumor M2 macrophages in the TME into anti-tumor M1 macrophages, persistent and effective anti-cancer treatment efficacy is anticipated (Fig. 4D). In addition to these approaches, simultaneously inhibiting mTOR, which exerts immunosuppressive effects on both innate and adaptive immunity, is expected not only to effectively eliminate cancer but also to potentially provide long-term benefits by reducing resistance to cancer treatment and preventing cancer recurrence.

### Advanced drug delivery systems

Regarding the effective delivery of anti-cancer drugs to CSCs, which often reside in the inner parts of tumor tissues, various drug delivery platforms are under development [297]. Since cancer cells typically originate from CSCs, primarily located at the center of the tumor mass, many drugs struggle to reach these CSCs, resulting in the failure of CSC eradication [298].

Recently, the development of drug delivery systems has involved the use of viruses, nanoparticles, and nanovesicles. Among viral delivery systems, adenovirus is a well-known carrier [299, 300]. However, adenovirus-based delivery faces challenges related to low efficacy and high toxicity that need to be addressed [282]. Viruses can also be employed to deliver microRNAs that target and block genes involved in CSC resistance and relapse [301]. Notably, microRNAs such as miR-15a and miR-16-1 are known for their tumor-suppressive properties [302, 303].

Nanoparticle therapeutics have demonstrated enhanced efficacy while simultaneously reducing side effects, making nanoparticle-based anti-cancer drug delivery systems promising for cancer treatment [304]. Although there are a number of nanoparticle types depending upon their components and ingredients, generally using the nanoparticles allows for targeted drug localization in tumors, resulting in increased cellular uptake of anti-cancer drugs [305, 306]. Consequently, not only pre-clinical studies but also clinical investigations have been conducted to advance cancer treatment using various types of nanoparticles [307, 308].

Nanovesicles include both extracellular vesicles (EVs) which are spontaneously generated by cells, and cell-derived nanovesicles (CDNVs) which are generally produced using serial extrusion methods [309]. Since the nanovesicles are not only easy to prepare at a large scale but also suitable for drug loading, they have emerged as promising carriers for anti-cancer therapy [310, 311]. Furthermore, leveraging the intrinsic characteristics of the source cell, nanovesicles can be applied to various types of cancer cells, with potential for expansion into immunotherapy, gene therapy, and cell therapy when derived from suitable source cell types [312].

The development of advanced drug delivery systems such as viruses, nanoparticles, and nanovesicles represents a promising frontier in overcoming the challenges of targeting CSCs within tumor tissues (Fig. 4E). These systems not only enhance drug delivery efficiency but also alleviate the toxicity associated with traditional treatments. Considering the unique properties of each delivery platform, researchers are advancing towards more effective and targeted anti-cancer treatments, potentially transforming the landscape of cancer treatment by improving outcomes and patient quality of life.

## Conclusions

Cancer is a highly complex disease to treat, given its various causes and symptoms that depend on the type of cancer and its location of occurrence [313]. Chemotherapy, a common method of tumor treatment, faces limitations due to drug resistance, necessitating the development of improved cancer therapy approaches [314, 315]. A range of hypotheses related to cancer development and regeneration have emerged, with one prominent strategy focusing on eliminating CSCs by targeting crucial pathways associated with them [202]. CSCs, akin to normal stem cells, possess the ability to differentiate into tumor cells and exhibit self-renewal ability [148]. In CSCs, several representative signaling pathways are involved in maintaining pluripotency and resistance to therapeutics. Consequently, numerous studies have been conducted, encompassing in vitro, in vivo and clinical trials. Among these pathways, the mTOR pathway is particularly noteworthy for its significant role in activating not only stem cells but also immune cells within cancer tissues [28, 29]. In 1999, the representative mTOR inhibitor, rapamycin, received FDA approval as an immune suppressor that reduces the activation of T helper 17 cells (Th17 cells), which are implicated in inflammation and immune homeostasis [316]. Subsequently, with the discovery of the association between mTOR signaling and cancer, FDA-approved mTOR inhibitors have been employed in clinical trials for cancer treatment, while various other mTOR inhibiting molecules are currently under development [28]. Table 2 presents clinical trials for anti-cancer therapy using mTOR inhibitors categorized by cancer types.

**Fig. 5:**
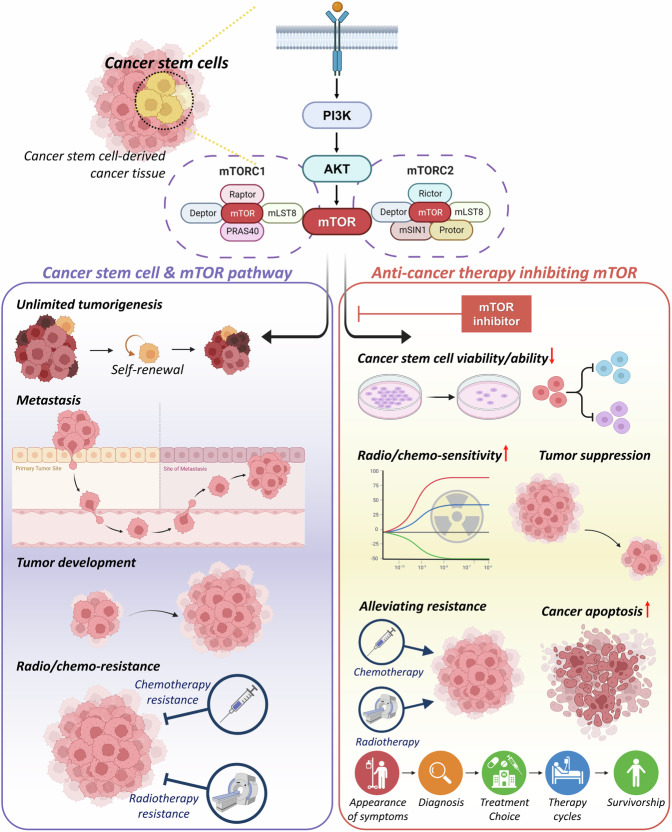
Anti-cancer therapy targeting CSCs through mTOR signaling pathway. The mTOR pathway-mediated cellular mechanisms in CSCs are summarized (purple box in left), and the results of mTOR inhibition is also described (pink box in right). In CSCs, mTOR signal induce unlimited tumorigenesis, metastasis [19–23], tumor development, and radio/chemo-resistance [39, 40], resulting in relapse of cancer and failure in anti-cancer therapy. However, by inhibiting mTOR signaling pathway, CSC viability and ability decrease [89, 95, 97–99, 102, 109], radio/chemo-sensitivity increase, alleviating resistance [100], tumor suppression is induced, resulting in cancer cell apoptosis [87, 88, 90–92, 108, 111]. Therefore, elimination of the CSCs using mTOR inhibition is a powerful strategy with potentials in anti-cancer therapy.

As described in this manuscript and the tables, research involving the treatment of mTOR inhibitors alone or in combination with other anticancer drugs on various types of CSCs has demonstrated that inhibiting the mTOR pathway suppresses not only CSC differentiation into tumor cells but also intrinsic stem cell properties, such as self-renewal and uncontrolled proliferation [161–163, 178, 182, 197, 198]. Additionally, mTOR-inhibited CSCs have shown increased apoptosis, resulting in decreased metastasis [167, 169, 189, 200]. The CSC-targeting therapy via mTOR inhibition has shown enhanced anti-cancer efficacy, offering hope for addressing existing limitations in cancer treatment, such as drug resistance caused by the active CSCs (Fig. 5) [159, 184, 317–319]. However, it is important to note that the mTOR pathway is involved in various cellular activities, not only CSCs but also in normal cells. Therefore, strategies involving mTOR inhibition require careful consideration to avoid potential side effects [320, 321]. Nonetheless, the elimination of CSCs using mTOR inhibition remains a potent approach in anti-tumor therapy, and advanced tumor-targeting techniques with specificity are required for enhanced efficiency.

## Data Availability

The published article includes all data sets generated/analyzed for this study.
